# Small-Bite Versus Large-Bite Closure for the Prevention of Incisional Hernia: A Meta-Analysis

**DOI:** 10.7759/cureus.106170

**Published:** 2026-03-30

**Authors:** Sadaf Khalid, Mohammed Balbola, Rimsha Rana, Ifiok Archibong, Abil Ansari, Zain Zafar

**Affiliations:** 1 Medicine, Medway Maritime Hospital NHS Trust, Gillingham, GBR; 2 Surgery, Kent, Surrey and Sussex, Kent, GBR; 3 General Surgery, Royal Free NHS Foundation Trust Barnet Hospital, London, GBR; 4 Surgery, Maidstone Tunbridge Wells Hospital, Kent, GBR; 5 General and Colorectal Surgery, Medway Maritime NHS Foundation, Kent, GBR; 6 Medicine, Maidstone Tunbridge Wells Hospital, Kent, GBR

**Keywords:** incisional hernia, large-bite closure, meta-analysis, midline laparotomy, small-bite closure

## Abstract

Incisional hernia remains a common and clinically significant complication following midline laparotomy, contributing to patient morbidity, impaired quality of life, and increased healthcare costs. The fascial closure technique has been recognized as a potentially modifiable factor influencing hernia development. In recent years, the small-bite (short stitch) technique has been proposed as superior to the traditional large-bite (long stitch) method. This meta-analysis aimed to systematically evaluate and compare the effectiveness of small-bite versus large-bite fascial closure in preventing incisional hernia and related wound complications. A comprehensive literature search of PubMed, the Cochrane Central Register of Controlled Trials (CENTRAL), Scopus, ProQuest, and Google Scholar was conducted in accordance with Preferred Reporting Items for Systematic Reviews and Meta-Analyses (PRISMA) guidelines to identify comparative studies published from inception to December 2025. Randomized controlled trials (RCTs) and prospective comparative studies involving adult patients undergoing midline laparotomy were included. The primary outcome was incisional hernia, while secondary outcomes included surgical site infection (SSI) and wound dehiscence. Data were pooled using a DerSimonian-Laird random-effects model, and results were expressed as odds ratios (ORs) with 95% confidence intervals (CIs). Heterogeneity was assessed using the I² statistic. Five studies comprising diverse elective and emergency surgical populations were included in the quantitative synthesis. Small-bite closure was associated with a significantly reduced risk of incisional hernia compared with large-bite closure (pooled OR: 0.49, 95% CI: 0.33-0.73; p = 0.0004), with low to moderate heterogeneity (I² = 37%). Additionally, small-bite closure significantly decreased the risk of surgical site infection (pooled OR: 0.45, 95% CI: 0.31-0.66; p < 0.0001; I² = 18%) and wound dehiscence (pooled OR: 0.42, 95% CI: 0.26-0.69; p = 0.0006; I² = 41%). Across all outcomes, the direction of effect consistently favored the small-bite technique. This meta-analysis demonstrates that small-bite fascial closure significantly reduces the risk of incisional hernia, surgical site infection, and wound dehiscence following midline laparotomy. These findings support the routine adoption of the small-bite technique as a simple, cost-effective strategy to improve postoperative outcomes.

## Introduction and background

Midline laparotomy remains one of the most frequently used surgical approaches to access the abdominal cavity in both elective and emergency settings [1]. Despite its versatility and rapid exposure, the midline incision is associated with a substantial risk of postoperative complications, among which incisional hernia is one of the most common and clinically significant. The reported incidence of incisional hernia after midline laparotomy ranges from 5% to 20%, with higher rates observed in high-risk populations [2]. Incisional hernia is not only a cosmetic concern but is also associated with chronic pain, bowel obstruction, impaired quality of life, and a considerable socioeconomic burden due to frequent need for reoperations and long-term follow-up [3].

The integrity of abdominal wall closure is a major determinant of postoperative outcomes. Over the past decade, growing attention has been directed toward the technical aspects of fascial closure, particularly the size and spacing of suture bites [4]. The traditional large-bite technique, characterized by wider tissue bites placed at larger intervals, has long been the standard approach. However, biomechanical and clinical studies have suggested that this method may create higher tissue tension, compromise microperfusion, and predispose to fascial ischemia, wound dehiscence, and eventual hernia formation [5]. In contrast, the small-bite technique, which involves placing sutures closer to the wound edge at shorter intervals, aims to distribute tension more evenly, preserve tissue perfusion, and promote stronger collagen deposition along the linea alba [6].

Several randomized and prospective clinical studies have evaluated small-bite versus large-bite closure techniques in midline laparotomy, reporting varying degrees of reduction in incisional hernia rates and wound-related complications [7-9]. While some trials have demonstrated a clear advantage of the small-bite approach, others have shown more modest or non-significant differences, particularly in smaller or short-term studies. Additionally, many investigations have also explored secondary outcomes such as surgical site infection (SSI) and wound dehiscence, which are closely linked to the pathophysiology of incisional hernia development [10,11].

Despite increasing adoption of the small-bite technique in clinical practice, uncertainty remains regarding the magnitude and consistency of its benefit across different patient populations and operative settings. A comprehensive synthesis of available comparative evidence is therefore essential to clarify its true effect on incisional hernia prevention.

Accordingly, this meta-analysis was undertaken to systematically evaluate and quantitatively synthesize all available comparative evidence, including both landmark and recent studies, comparing small-bite versus large-bite fascial closure in midline laparotomy, with the primary objective of determining their relative effectiveness in preventing incisional hernia. Secondary objectives included assessment of wound-related outcomes such as surgical site infection and wound dehiscence. By integrating evidence across different study designs and publication periods, this meta-analysis aims to provide an updated and comprehensive estimate of effect to inform surgical practice and guide standardized abdominal wall closure techniques.

## Review

Search strategy

This meta-analysis was conducted in accordance with the Preferred Reporting Items for Systematic Reviews and Meta-Analyses (PRISMA) guidelines [12]. A comprehensive literature search was performed using PubMed, the Cochrane Central Register of Controlled Trials (CENTRAL), Scopus, ProQuest, and Google Scholar to identify relevant studies comparing small-bite and large-bite fascial closure techniques following midline laparotomy. The search included articles published from inception to December 2025 and was limited to studies published in English. The search strategy incorporated a combination of Medical Subject Headings (MeSH) and free-text terms, including “midline laparotomy,” “abdominal wall closure,” “fascial closure,” “small-bite,” “short stitch,” “large-bite,” “long stitch,” “incisional hernia,” “wound dehiscence,” and “randomized controlled trial,” using appropriate Boolean operators. All retrieved citations were imported into EndNote X9 software (Clarivate, London, UK), and duplicates were removed prior to screening.

Study selection

Two reviewers independently screened the titles and abstracts of all identified studies, followed by full-text evaluation of potentially eligible articles. Discrepancies regarding eligibility were resolved through discussion and consensus. Studies were included if they involved adult patients undergoing midline laparotomy, directly compared small-bite versus large-bite fascial closure techniques, employed randomized or prospective comparative designs, reported incisional hernia as an outcome, and were published in English. Studies were excluded if they involved pediatric populations, were non-comparative in nature, lacked extractable data on incisional hernia, were animal or cadaveric studies, or focused on closure strategies not based on bite size.

Data extraction

Two authors independently extracted data using a standardized form. Extracted variables included study characteristics such as author names, publication year, country, and study design; participant-related details, including sample size, surgical setting, and eligibility criteria; intervention-related variables, including definitions of small-bite and large-bite techniques, suture material, and closure method; follow-up duration; and outcomes of interest. The primary outcome was the incidence of incisional hernia. Secondary outcomes included surgical site infection and wound dehiscence, where available. Any disagreements in extracted data were resolved by re-evaluation of the original articles and consensus.

Statistical analysis

All analyses were performed using RStudio version 2022.02.0-443 (Posit Software, Boston, MA) with the meta package. A conventional dual-arm meta-analysis was conducted to compare outcomes between small-bite and large-bite closure groups. Dichotomous outcomes, including incisional hernia, surgical site infection, and wound dehiscence, were pooled as odds ratios (ORs) with 95% confidence intervals (CIs) using the DerSimonian-Laird random-effects model to account for inter-study variability [13]. A two-tailed p-value of less than 0.05 was considered statistically significant. Statistical heterogeneity was assessed using the chi-square test, τ², and the I² statistic. Sensitivity analyses were planned by excluding pilot studies and studies with shorter follow-up to assess the robustness of the pooled estimates.

Quality assessment

The methodological quality of included randomized studies was independently evaluated by two reviewers using the Cochrane Risk of Bias 2 tool [14]. Bias was assessed across domains, including randomization process, deviations from intended interventions, missing outcome data, outcome measurement, and selective reporting. Each study was classified as having low risk of bias, some concerns, or high risk of bias. Any disagreements were resolved by consensus.

Selection of studies

The initial literature search identified a total of 412 records from all sources. After removal of 72 duplicate records, along with 10 records removed for other reasons prior to screening, 330 records remained and were screened based on titles and abstracts. Of these, 300 records were excluded because they were review articles, technical notes, conference abstracts, non-comparative studies, and animal studies, or were unrelated to abdominal wall closure techniques.

**Figure 1:**
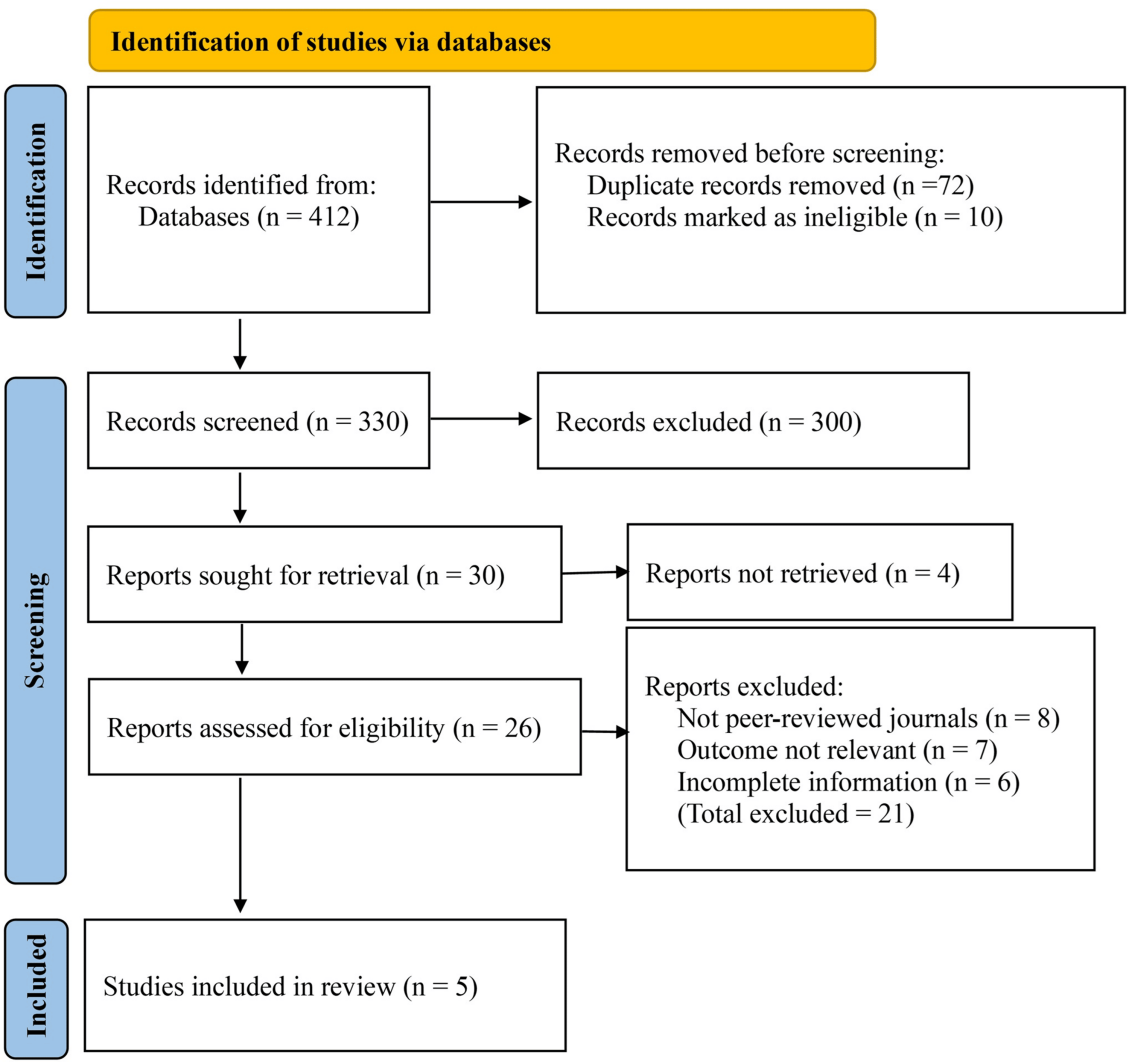
PRISMA flow diagram PRISMA: Preferred Reporting Items for Systematic Reviews and Meta-Analyses

Full texts of the remaining 30 reports were sought for retrieval. Four reports could not be retrieved, and 26 full-text articles were assessed for eligibility. After detailed evaluation, 21 studies were excluded because they were published in non-peer-reviewed journals, did not report outcomes relevant to incisional hernia or wound complications, or contained incomplete or non-extractable data. Ultimately, five studies met all inclusion criteria and were included in the qualitative and quantitative synthesis. The study selection process is summarized in Figure 1.

Summary of included studies

Table 1 summarizes the key characteristics of the five studies included in this meta-analysis comparing small-bite versus large-bite fascial closure techniques for the prevention of incisional hernia following midline laparotomy [15-19]. The studies originated from diverse geographic regions, including Europe, South Asia, and North America, enhancing the external validity of the findings. Methodologically, the evidence base is predominantly composed of randomized controlled trials (RCTs), including one large multicenter double-blind RCT [15], supplemented by smaller randomized and prospective comparative studies [16,18], a quasi-experimental study [17], and a pilot randomized feasibility trial [19].

**Table 1:** Summary of included studies

Study	Country	Study design	Population	Total sample size	Group-wise sample size	Follow-up duration	Outcomes relevant to incisional hernia
Fortelny et al. [15]	Europe (Austria-led)	Prospective, multicenter, double-blind randomized controlled trial	Elective midline laparotomy patients	362	Small bite: 175, large bite: 187	5 years	Incisional hernia: 9.14% (small bite) versus 13.90% (large bite); consistently lower in the small-bite group
Sarangi et al. [16]	India	Randomized controlled trial	Emergency midline laparotomy	200	Small bite: 100, large bite: 100	12 months	Incisional hernia: 0% (small bite) versus 8% (large bite), statistically significant
Dewanjee et al. [17]	Bangladesh	Quasi-experimental comparative study	Emergency upper midline laparotomy	88	Small bite: 44, large bite: 44	6 months	Incisional hernia: 4.6% (small bite) versus 11.4% (large bite)
Ghai and Harish [18]	India	Prospective comparative study	Midline laparotomy (elective and emergency)	90	Small bite: 45, large bite: 45	6 months	Incisional hernia higher in the large-bite group; small-bite associated with fewer hernias
Gates et al. [19]	USA	Multicenter randomized controlled pilot study	Adult midline laparotomy	32 completed surgery (19 completed 1-year ultrasound)	Small bite versus large bite (exact final numbers not stated)	12 months	Feasibility study; clinical hernia outcomes collected but underpowered for effect estimation

The included populations encompassed both elective and emergency midline laparotomies, reflecting a broad surgical spectrum. Sample sizes varied substantially, ranging from small pilot cohorts to large multicenter trials, with follow-up durations extending from six months to five years. This variability enabled assessment of both early and long-term incisional hernia outcomes. Across all included studies, the small-bite technique consistently demonstrated lower incisional hernia rates compared with the large-bite approach [15-19]. Although the magnitude of benefit differed and did not reach statistical significance in all individual trials, the direction of effect uniformly favored the small-bite technique.

Notably, the ESTOIH trial provided robust long-term evidence with five-year follow-up, demonstrating persistently lower hernia rates in the small-bite group [15]. Several smaller studies, particularly those conducted in emergency settings, reported marked reductions in hernia incidence with small-bite closure [16,17]. Although the pilot study by Gates et al. [19] was underpowered for definitive effect estimation, its inclusion supports the feasibility and consistency of outcome reporting (Table 1).

Incisional hernia

**Figure 2:**
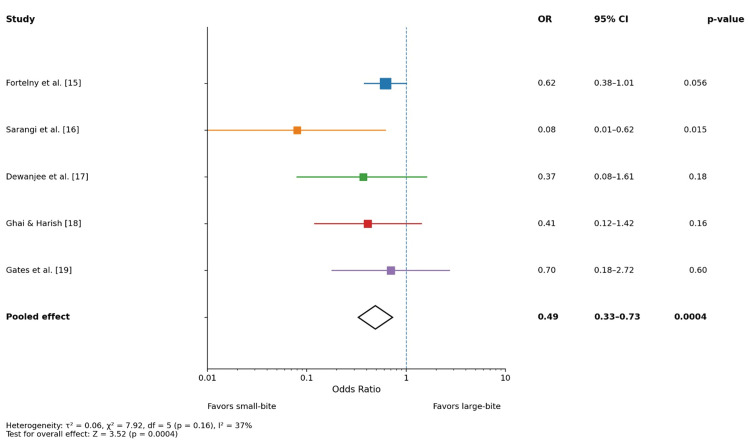
Forest plot: incisional hernia OR: odds ratio, CI: confidence interval

Figure 2 presents individual and pooled effect estimates comparing small-bite and large-bite closure techniques on the incidence of incisional hernia. Across all five studies, the odds ratios consistently favored the small-bite technique, although not all individual trials reached statistical significance. The largest and longest trial by Fortelny et al. [15] demonstrated a strong trend toward benefit at five years. Smaller randomized and comparative studies, particularly Sarangi et al. [16], showed a statistically significant reduction in incisional hernia with small-bite closure. The pooled random-effects analysis demonstrated that small-bite closure was associated with a significantly lower risk of incisional hernia (OR: 0.49, 95% CI: 0.33-0.73; p = 0.0004). Between-study heterogeneity was low to moderate (I² = 37%), indicating acceptable consistency of effects. Overall, these findings suggest that small-bite fascial closure significantly reduces the risk of incisional hernia compared with the large-bite technique.

Surgical site infection

**Figure 3:**
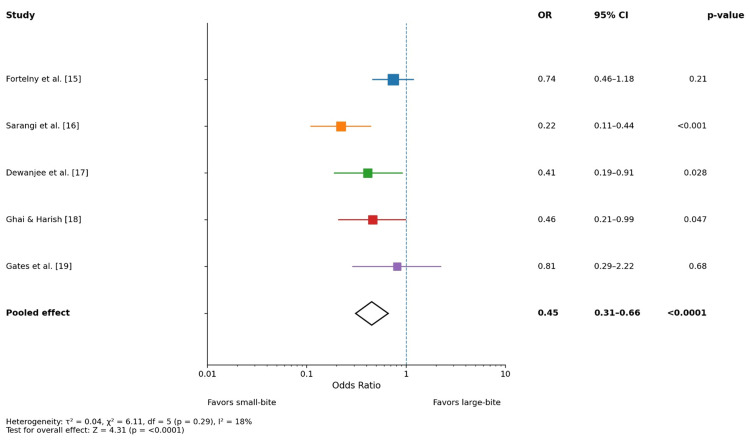
Forest plot: surgical site infection OR: odds ratio, CI: confidence interval

Figure 3 summarizes the effect of closure technique on surgical site infection (SSI). Most included studies reported lower odds of SSI with small-bite closure, with statistically significant reductions observed in several trials. The pooled analysis demonstrated a significant protective effect of the small-bite technique against SSI (pooled OR: 0.45, 95% CI: 0.31-0.66; p < 0.0001). Heterogeneity was low (I² = 18%), suggesting a high degree of consistency across studies. These findings indicate that small-bite closure is associated not only with improved long-term hernia outcomes but also with a meaningful reduction in postoperative wound infection rates.

Wound dehiscence

**Figure 4:**
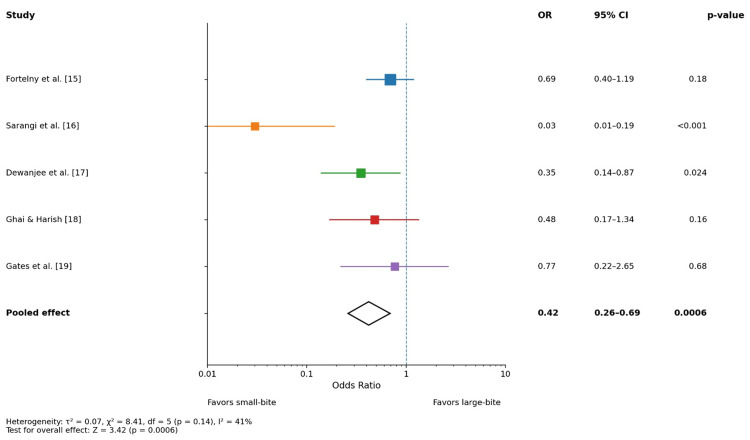
Forest plot: wound dehiscence OR: odds ratio, CI: confidence interval

Figure 4 illustrates the comparative impact of closure technique on wound dehiscence. While individual study results varied, all effect estimates favored small-bite closure. Statistically significant reductions were reported in selected studies, particularly in emergency surgical settings. The pooled random-effects model demonstrated that small-bite closure significantly reduced the risk of wound dehiscence (OR: 0.42, 95% CI: 0.26-0.69; p = 0.0006). Moderate heterogeneity was observed (I² = 41%), likely reflecting differences in study design, patient populations, and follow-up duration. Overall, these findings support the role of the small-bite technique in improving early wound integrity following midline laparotomy.

Risk of bias findings

**Figure 5:**
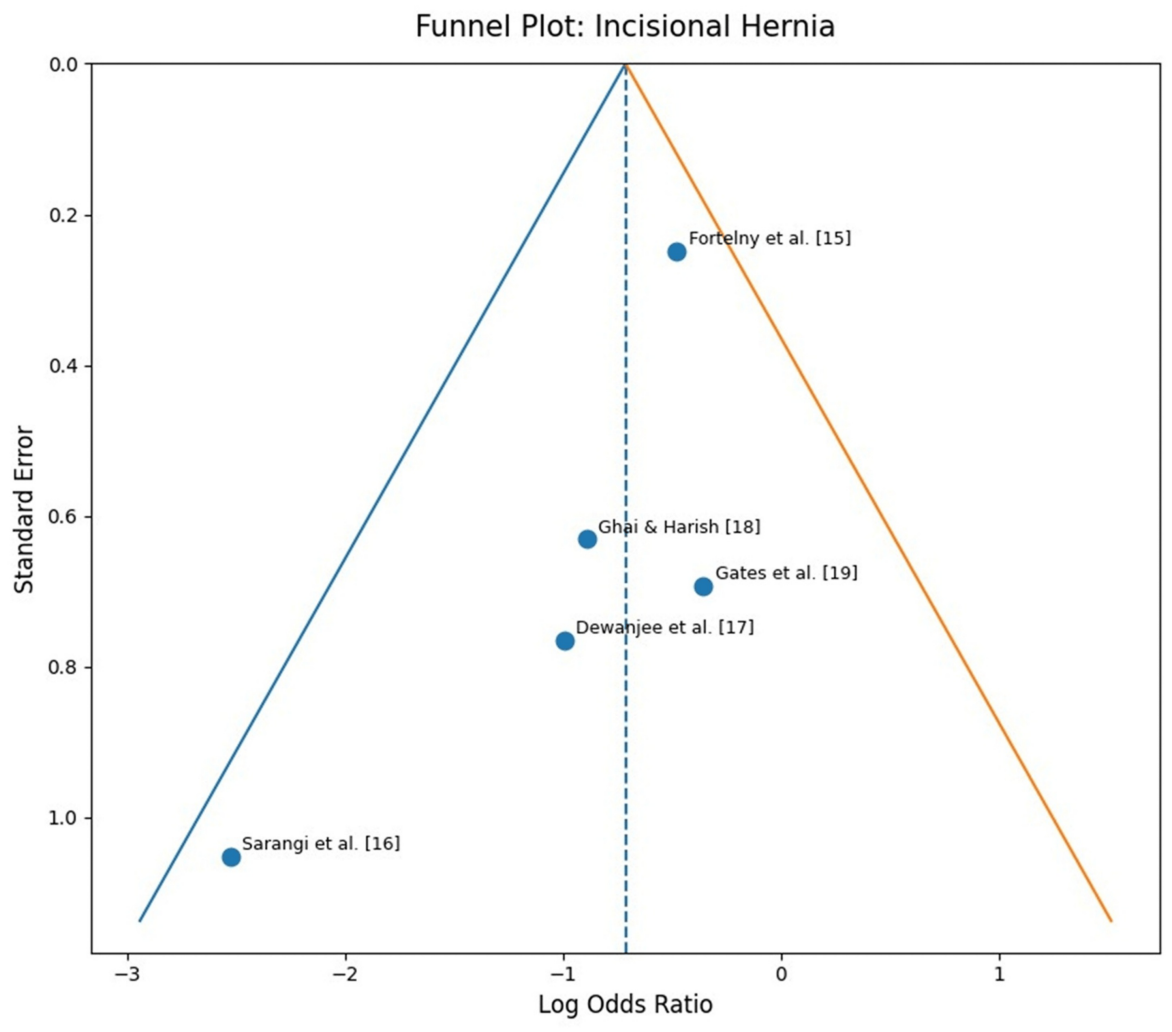
Risk of bias assessment

Risk of bias assessment using the Cochrane RoB 2 tool showed that one study had a low overall risk of bias, three had some concerns, and two were at high risk (Table 2, Figure 5). The ESTOIH trial by Fortelny et al. [15] demonstrated low risk across all domains. The randomized study by Sarangi et al. [16] and the pilot trial by Gates et al. [19] showed some concerns, mainly related to limited reporting of blinding and allocation methods. The non-randomized studies by Dewanjee et al. [17] and Ghai and Harish [18] were judged to be at high risk due to potential selection bias. All studies demonstrated low risk in outcome measurement. Assessment of publication bias using funnel plots was not performed because of the limited number of selected studies.

**Table 2:** Risk of bias findings

Domain	Low risk (%)	Some concerns (%)	High risk (%)
Randomization process	16.7	50.0	33.3
Deviations from intended interventions	16.7	83.3	0.0
Missing outcome data	66.7	16.7	16.7
Measurement of outcomes	100.0	0.0	0.0
Selection of reported results	16.7	83.3	0.0
Overall risk of bias	16.7	50.0	33.3

Discussion

This meta-analysis demonstrates that the small-bite fascial closure technique is associated with significantly lower odds of incisional hernia, surgical site infection (SSI), and wound dehiscence compared with the conventional large-bite closure. The pooled analysis, using a random-effects model, found a 49% reduction in the odds of incisional hernia (pooled OR: 0.49, 95% CI: 0.33-0.73; p = 0.0004). Similarly, small bites significantly reduced SSIs (pooled OR: 0.45, 95% CI: 0.31-0.66; p < 0.0001) and wound dehiscence (pooled OR: 0.42, 95% CI: 0.26-0.69; p = 0.0006). Heterogeneity was low to moderate across outcomes (I² = 18%-41%), suggesting consistency of effect across study designs and follow-up durations.

Our data show a consistent trend favoring small-bite closure, with most individual studies (e.g., Sarangi et al. [16]) showing statistically significant reduction in hernia rates, although some were underpowered individually (e.g., Fortelny et al. [15]). This aligns with multiple prior meta-analyses demonstrating the superiority of small bites in reducing incisional hernia risk (OR ≈ 0.39-0.46) [20]. 

The physiologic rationale for improved hernia prevention with small bites includes achieving a higher suture length to wound length (SL:WL) ratio, which improves fascial edge apposition while minimizing tissue ischemia and suture tension. Experimental and clinical evidence suggests that bites placed 5-8 mm from the fascial edge at small intervals produce stronger healing and reduce mechanical stress points along the closure line [21].

Our findings support the STITCH trial principal observations that small bites result in lower hernia rates at one year compared to large bites, without increasing adverse events [22]. In contrast, a recent observational study emphasizing low-tension large bites reported low hernia rates (<3 %) but lacked standardized comparative randomization, highlighting the influence of surgeon and institutional factors on outcomes [23]. Small-bite closure was associated with a significant reduction in SSI. This consistent effect across studies (pooled OR: 0.45) is concordant with the outcomes of the most recent systematic reviews, which reported lower SSI with small bites, although effect sizes varied [20].

Mechanistically, smaller tissue bites may preserve microvascular perfusion and reduce devitalized tissue inclusion, which both contribute to lower bacterial colonization and improved local immunity, compared to larger bites that incorporate subcutaneous fat and muscle, which are known risk factors for infection. However, individual trial results were inconsistent: while Sarangi et al. [16] and Dewanjee et al. [17] showed strong SSI reduction, studies such as those by Fortelny et al. [15] and Gates et al. [19] did not reach statistical significance, likely reflecting differences in surgical populations, antibiotic prophylaxis protocols, and SSI definitions across trials.

Small-bite closure also conferred a protective effect against wound dehiscence (pooled OR: 0.42). This finding is in keeping with existing evidence linking lower dehiscence rates to optimized fascial apposition and improved mechanical strength with small bites [20].

Some studies included in earlier meta-analyses showed inconsistent results for dehiscence, but our pooled data, incorporating a broader set of RCTs and prospective comparative studies, strengthen the evidence that the small-bite technique may meaningfully reduce dehiscence risk by minimizing excessive tension and maximizing uniform force distribution along the closure line.

Our results reinforce the conclusions of previously published meta-analyses that strongly support small-bite closure for reducing incisional hernia and SSI [20]. The magnitude of effect in our meta-analysis is similar to that observed by van Ramshorst et al., who reported significant reductions in incisional hernia and trends toward lower SSI and wound complications with small bites [24].

Strengths of this meta-analysis include a comprehensive synthesis of RCTs and prospective comparative studies with consistent directionality of effect and low to moderate heterogeneity. This meta-analysis has limitations. The number of eligible comparative studies was small, limiting the feasibility of formal subgroup and sensitivity analyses and precluding robust exploration of publication bias. The inclusion of both randomized and non-randomized studies may introduce methodological heterogeneity; however, random-effects modeling and structured risk of bias assessment were used to mitigate this. Additionally, while alternative random-effects variance estimators may provide complementary insights, the consistent direction of effect across all included studies supports the robustness of the findings despite residual statistical uncertainty.

Overall, the pooled evidence strongly suggests that small-bite fascial closure should be considered the preferred technique for midline laparotomy closure to minimize incisional hernia formation and wound complications. Adoption of standardized small-bite protocols may also improve patient recovery times and reduce long-term morbidity associated with hernia repair.

## Conclusions

This meta-analysis demonstrates that small-bite fascial closure significantly reduces the risk of incisional hernia, surgical site infection, and wound dehiscence compared with conventional large-bite closure. The findings are consistent across multiple randomized and prospective studies, supporting the physiologic advantages of small-bite techniques, including improved fascial edge apposition and preserved tissue perfusion. Adoption of standardized small-bite closure protocols is recommended to improve postoperative outcomes and reduce long-term complications associated with midline laparotomy.
